# Assessment of arsenic and polycyclic aromatic hydrocarbon (PAH) exposures on immune function among males in Bangladesh

**DOI:** 10.1371/journal.pone.0216662

**Published:** 2019-05-16

**Authors:** Faruque Parvez, Fredine T. Lauer, Pam Factor-Litvak, Xinhua Liu, Regina M. Santella, Tariqul Islam, Mahbubul Eunus, Nur Alam, Golam Sarwar, Mizanour Rahman, Habibul Ahsan, Joseph Graziano, Scott W. Burchiel

**Affiliations:** 1 Department of Environmental Health Sciences, Mailman School of Public Health, Columbia University, New York, NY, United States of America; 2 University of New Mexico College of Pharmacy, Department of Pharmaceutical Sciences, Albuquerque, NM, United States of America; 3 Department of Epidemiology, Mailman School of Public Health, Columbia University, New York, NY, United States of America; 4 Department of Biostatistics, Mailman School of Public Health, Columbia University, New York, NY, United States of America; 5 University of Chicago Field Research Office, Bangladesh; 6 Department of Health Studies, University of Chicago, Chicago, IL, United States of America; University of Rajshahi, BANGLADESH

## Abstract

Arsenic and polycyclic aromatic hydrocarbons (PAH) are environmental pollutants to which people around the world are exposed through water, food and air. In mouse and *in vitro* studies of human cells, both of these chemicals have been shown to modulate the immune system. In some experimental studies, a synergistic disruption of immune function was observed by a combined exposure to arsenic and PAH. However, a joint effect of arsenic and PAH on immune function has not been studied in humans. We have conducted an epidemiological investigation to examine effects of chronic arsenic and PAH exposures on immune function. We assessed T-cell proliferation (TCP) and cytokine production of anti-CD3/anti-CD28 stimulated lymphocytes in human peripheral blood mononuclear cells (HPBMC) among 197 healthy men enrolled to the Health Effects of Arsenic Longitudinal (HEALS) cohort in Bangladesh. By design, approximately half were active smokers and the rest were never smokers. Our analyses demonstrated that IL-1b, IL-2, IL-4 and IL-6 were significantly stimulated as a function of urinary arsenic levels in models adjusted for age, body mass index (BMI), smoking status and PAH-DNA adducts. After correcting for false detection rate (FDR), only IL-1b remained statistically significant. We found a U-shaped dose response relationship between urinary arsenic and IL-1b. On the other hand, PAH-DNA adducts were associated with an inhibition of TCP and appeared as an inverted U-shape curve. Dose response curves were non-monotonic for PAH-DNA adduct exposures and suggested that cytokine secretion of IFNg, IL-1b, IL-2, IL-10 and IL17A followed a complex pattern. In the majority of donors, there was a trend towards a decrease in cytokine associated with PAH-DNA adducts. We did not observe any interaction between urinary arsenic and PAH-DNA adducts on immune parameters. Our results indicate that long-term exposures to arsenic and PAH have independent, non-monotonic associations with TCP and cytokine production.

## Introduction

In Bangladesh, exposure to arsenic has been associated with numerous adverse health outcomes [1, 2]. In our cohort, exposure to arsenic is associated with cancer [3, 4], cardiovascular disease [5, 6], and lung disease [7–9] in adults, and with cognitive impairment in children [10, 11]. Other studies suggest that arsenic also increases the risk of upper airway infections in children [12–14], which is consistent with studies in arsenic exposed animal models and systemic viral infections [15].

Because the immune system plays an important role in protecting against cancers and infection, the purpose of the present study was to assess the effects of chronic arsenic exposures on functional measures of the human immune system, including TCP and cytokine production, measured in HPBMC from males living in Bangladesh. We also examined the role of PAH exposure, as it is also associated with immune modulation and our previous work in mouse models demonstrated that there may be important interactions between PAHs and arsenic [16, 17]. We examined the influence of PAH exposure, as indicated by *in vivo* PAH-DNA adducts, on *ex vivo* immune function and the potential interactions between PAH-DNA adducts and urinary arsenic in statistical models.

While arsenic has been shown to suppress human immune cells examined *in vitro* [18–22], there have been only a few previous studies examining the effects of arsenic on the human immune system following *in vivo* exposure. Biswas et al. [23] found that drinking water arsenic was associated with suppression of TCP and cytokine production in HPBMC among individuals living in an arsenic endemic area in West Bengal, India. Similarly, Soto-Pena et al. [24] reported a decrease in the TCP response, a decrease in IL-2 production, and a slight decrease in circulating CD4 cells in children living in Mexico exposed to arsenic. Banerjee et al. [25] found the macrophages derived from HPBMC in donors exposed to arsenic in drinking water had altered cell morphology, activation markers, and phagocytic activity. Following developmental exposures, Raqib et al. [26] found that total serum immunoglobulins were elevated and vaccine responses were attenuated in boys exposed to arsenic. Prenatal exposure to arsenic was also associated with decreased cell-mediated immunity in Bangladeshi children [27]. Thus, there is evidence that arsenic exposure alters immune markers and immune function, suggesting that HPBMC examined *ex vivo* may be useful to examine mechanisms of immunomodulation.

The overall aim of this study was to assess associations between long-term chronic arsenic and PAH exposure and changes in immune function in HPBMC among males in Bangladesh and to examine possible interactions between these two exposures on these outcomes. We chose a variety of immune function parameters that are primarily associated with peripheral blood lymphocyte function because previous work *in vitro* has shown that T lymphocytes are highly sensitive to arsenic exposure.

## Methods

### Recruitment of study participants

Participants were recruited and consented as described by Parvez et al. [28]. Briefly, we recruited subjects from the Health Effects Arsenic Longitudinal Study (HEALS) [1]. A list of potential participants for the study was generated from the HEALS central database based on arsenic exposure history, age and smoking status. Since one of the goals of the study was to examine a joint effects of arsenic and PAH, we recruited half of the participants with drinking water > 50 μg/L arsenic and the rest <50 μg/L. Similarly, half of the participants were current smokers and half were never smokers. Adult healthy men age 35–65 years were included in the study. Smoking among females is very low (<3%) in Bangladesh, therefore we recruited only males in this study. Persons with illnesses related to immune dysfunction and/or taking medications that might impact immune function, including those for cardiovascular disease and diabetes, were not eligible.

The study protocol was approved by the Institutional Review Board of Columbia University and ethical clearance was obtained from the Bangladesh Medical Research Council (BMRC). The Institutional Review Board of the University of New Mexico approved a protocol for the analysis of the samples. Informed consent was obtained from all participants either in written or verbal. All the materials, including consent forms, were translated into Bengali and back translated in to English. For the participants unable to read or write the informed consent was read to them and all procedures were explained, in the presence of a witness, in simple language by a village health worker who is from the area.

We identified 317 eligible participants, of whom 267 visited the study clinic and completed all study procedures. Of the 267 participants, blood samples were obtained and analyzed from 200. Because some samples did not have an adequate viability or number of cells to conduct all of the study assays, 197 samples were assayed for TCP and cytokine production.

### Collection and cryopreservation of PBMC

A detailed procedure for the isolation and cryopreservation of HPBMC has been published elsewhere by our group [29]. Briefly, blood samples were collected from the arm of consented donors into vacuum blood tubes containing sodium heparin as anticoagulant. Blood was then diluted 1:1 with Dulbecco’s phosphate-buffered saline (Sigma-Aldrich, D8537) without calcium or magnesium (DPBS^-^) at room temperature. Twenty-eight ml of diluted blood was then layered over 20 ml Fico/Lite-LymphoH (Atlanta Biologicals, I40150) without mixing the layers in a 50 ml centrifuge tube (all reagents and blood were at room temperature). The samples were then centrifuged for 30 min at 400xg with the brake off. Following centrifugation, the mononuclear cell layer at the interface of the Fico/Lite layer was collected into a 15 ml tube and mixed with 15 ml cold DPBS^-^. Samples were centrifuged for 10 min at 250xg, the supernatant was aspirated from the cell pellet and cells were re-suspended with cold DPBS^-^. Multiple cell pellets from a single donor were combined into one 15 ml tube and washed two more times with cold DPBS^-^ as described previously. Cells were resuspended and cryopreserved at approximately 2x10^7^ cells/ml using Freezing media A + B (Athena Enzyme Systems, 0406) at 1:1 ratio and transferred into 2 ml cryovials. The cryovials were placed at -80°C for at least 24 hr after which time they were transferred to liquid nitrogen for storage.

### Transport of cryopreserved HPBMC

Cryopreserved HPBMC samples were transported by air to the United States using Dry Shippers from Cryoport, Inc. (Irvine, CA). The Dry Shippers allowed the samples to remain frozen (< -180°C) for more than seven days. Once the shipper and samples arrived at the University of New Mexico, they were transferred into liquid nitrogen until thawed for assays.

### Thawing of HPBMC

Each sample was thawed quickly in a 37°C water bath by swirling the cyrovial around in the water. Just as the sample thawed, it was transferred using sterile technique to 10 ml of RPMI- HEPES modified (Sigma- Aldrich,R5886) containing 10% FBS, 1% of 200 mM L-glutamine (Gibco, 25030–081) and 1% of 10,000 U/ml penicillin and 10,000 μg/ml streptomycin (Gibco, 15140–122), referred to here on as complete media. The sample was centrifuged at 230xg at RT, media was aspirated, the cell pellet was resuspended in 5 ml complete media, this wash step was repeated and the remaining cell pellet was resuspended in 3 ml complete media. Samples were counted on the Nexcelom Cellometer Auto 2000 Cell Viability Counter using acridine orange and propidium iodide (AO/PI; Nexcelom Bioscience, CS2-0106) to discern live HPBMC from red blood cells (RBC) and dead HPBMC. Cells were brought up to a concentration of 2x10^6^ cell/ml in complete media. HPBMC were assayed for TCP and cytokine production as outlined below.

The preparation, cryopreservation, and shipping of samples in gas phase nitrogen (-180°C) are all critical steps, as well as the thawing and testing of samples to ensure that all sample had greater than 80% viability at the time of testing.

### T cell proliferation (TCP) assay

Stimulation of HPBMC was carried out by addition of a combination of immobilized anti-human CD3 [clone OKT3 functional grade eBiosciences; 16-0037-85] and solubilized anti-human CD28 [clone CD28.2 functional grade eBiosciences, 16-0289-85]. First, 100 μl of 0.5 μg/ml anti-hu CD3 (in DPBS^-^) was placed into each assay well of a tissue culture treated, flat-bottom, 96 well plate; the plate was then held in a humidified, 37°C, 5% CO_2_ incubator for at least 2 hr. After 2 hr and just prior to use, the plate was washed by twice adding 200 μl DPBS^-^ then drawing off the DPBS. Cells were plated at 1x10^5^ cells/well in replicates of six and 20 μl of 20 μg/ml anti-human CD28 was added to each well to yield a final concentration of 2 μg/ml in the well. Plates were then incubated for 72 hr in a humidified incubator at 37°C with 5% CO_2_. A “no mitogen” control was used to evaluate background stimulation using media in place of the mitogen (anti-CD3/anti-CD28). After 72 hours, cultures were pulsed with 1 μCi/well tritiated (^3^H) thymidine and returned to the incubator for an overnight incubation (approximately 18 hr). Following incubation, cells were harvested onto angel hair filters using a Brandel 96 well harvester (Gaithersburg, MD). Filters were air-dried for at least 1.5 hr at RT, then placed into scintillation vials containing scintillation fluid. Vials were counted on a Beckman Coulter LS6500 Multipurpose Scintillation Counter for 1.5 min per sample, data is reported as counts per minute (CPM).

### Meso scale discovery (MSD) multiplex electrochemiluminescence immunoassay for cytokine analysis

Cells were plated at 1x10^5^ cell/well and stimulated with anti-CD3/anti-CD28 (as described above) or with media as an unstimulated control. Plates were placed in a humidified, 37°C, 5% CO_2_ incubator for 24 hr or 72 hr. Replicate supernatant samples were collected into a sterile microcentrifuge tube that were then aliquoted and stored at -80°C until assayed. A Meso Scale Discovery (MSD) V-Plex Proinflammatory Panel 1 (Human) kit was used in conjunction with the MSD QuickPlex SQ 120 plate reader to analyze IFNg, IL-2 after 24 hr stimulation and IL-1b, IL-4, IL-6, IL-10 and TNFa after 72 hr stimulation. IL-8 (24 hr stimulation) and IL-17A (72 hr stimulation) analyses were carried out using IL-8 or IL-17A V-Plex, single spot (Human) kits by MSD. Stimulated supernatants were diluted 1:400 for 24h (IFN-g, IL-2 and IL-8), 1:100 for 72h (IL-1b, IL-4, IL-6, IL-10, TNF-aand IL-17A); unstimulated supernatants were analyzed undiluted for all cytokines except IL-8 which was diluted 1:400. All of the dilutions for the cytokine supernatants were carried out in complete media. Calibrator dilutions (8-point standard curve) were prepared at 1:4 dilutions following kit instructions. Cytokine concentrations were reported in pg/ml supernatant. For analytical purposes, samples falling below the fit curve or detection range were considered non-detectable and were assigned a value of one-half the lower detection limit for the assay.

### Water As, Urine collection, and UAs, and creatinine assays

Spot urine samples were collected in 50 mL acid-washed tubes and stored at −80°C until shipped to Columbia University on dry ice for analysis. Water As (WAs) and total urinary arsenic (UAs) measurements were performed by graphite furnace atomic-absorption spectrophotometry (GFAAS) using a Perkin-Elmer Analyst 600 graphite furnace system as previously described [30]. The detection limit for WAs and UAs was 2 μg/L. In the HEALS Cohort, there is a very high intra-correlation coefficient between WAs and UAs (0.64 95% CI: 0.63–0.65). Analysis of urinary creatinine was based on a colorimetric method based on the Jaffe reaction. Total urinary arsenic adjusted for creatinine was reported as μg As/g Cr (UAs/Cr). UAsCr was used for correlations with immune endpoints because it was a stronger indicator of As exposure at the time that blood samples were drawn for immune analyses. The contribution of dietary sources to total UAs was very low, with the mean of arsenobetaine and arseno choline detected in urine of 3.5%.

### Polycyclic aromatic hydrocarbon-DNA (PAH-DNA) analysis

PAH diol epoxide-DNA adducts were analyzed by competitive ELISA, using methods described previously [31]. Briefly, 96 microwell plates coated with 2 ng of benzo(a)pyrene diol epoxide (BPDE)-I-DNA (5 adducts/10^3^ nucleotides) and rabbit antiserum #29 [32] were used with BPDE-DNA as a standard. DNA was isolated from frozen HPBMC according to a standard procedure using phenol/chloroform/isoamyl alcohol. DNA was assayed for PAH-DNA adducts after sonication and denaturation by laboratory technicians blinded to exposure status. For analytical purposes, those samples with <15% inhibition are considered non-detectable and assigned a value of 1 adduct/10^8^ nucleotides, an amount midway between the lowest positive value and zero. A 5% blinded duplication was carried out using those subjects with the most DNA available. As an additional quality control, a DNA sample from an animal treated with BP was also assayed with each sample batch.

### Statistical analysis

The total number of participants was 197. Of those, 16 were excluded due to missing data on PAH-DNA adducts, for a total sample size of 181. In preliminary analyses, we calculated summary statistics for all variables. Spearman correlation coefficients were used to describe bivariate associations among the quantitative variables. Scatterplots were used to describe the preliminary relationships between the exposures of interest (e.g. urinary arsenic, PAHs) and each immune parameter, and thus inform the shape of the statistical model. We imputed the missing value for body mass index (BMI) for one participant based on a regression model for BMI with predictors of age and smoking status. Age, BMI and smoking status were included as covariates in all statistical models. To reduce the impact of extreme variables and/or meet model assumptions, we transformed variables with right skewed distributions; thus, most of the outcome variables were transformed using log, square root and cube root transformations.

We used generalized additive models (GAM) to evaluate possible non-monotonic relationships between each immune marker, as the outcome, and UAs/Cr (UAs adjusted for creatinine) and PAH (PAH-DNA adducts), as the exposures. GAMs are a flexible set of models and allow for both a parametric component of exposure (which assesses a linear relationship) and non-parametric components, which assesses non-monotonicity. Based on the results of the GAM models, we fit linear models for the immune parameter outcomes with the most parsimonious polynomials of the exposure variables to best describe the patterns of the associations. In these models, we used the likelihood ratio test to detect the overall effect of exposure (linear and polynomial terms). We calculated the change in R-squared to demonstrate the overall effect size for the linear and polynomial terms of the exposure as the interpretation of the estimated regression coefficients is difficult due to the transformations of the outcome variables.

To examine whether UAs/Cr modified the association between PAH-DNA adducts and any immune parameter, we stratified the sample by median of UAs/Cr and fit linear models, as suggested by the GAM. The Wald test was used to detect differences in the coefficients of PAH-DNA adducts variables between UAs/Cr strata above and below the median.

To adjust for multiple testing on 10 immune parameters, we used Benjamini and Hochberg method to control for false discovery rate (FDR) in multiple tests. The FDR is a method of conceptualizing the rate of type I errors in null hypothesis testing when conducting multiple test. These procedures are designed to control the expected proportion of rejecting null hypotheses that are incorrect rejections. In these analyses, we repeatedly used statistical test to determine whether the estimated associations between UAs/Cr or PAH-DNA adducts and the immune parameters were statistically significant. To control for the potential of false discovery, we used the Benjamini and Hochberg procedure.Relevant associations were those having p-values as well as FDR <0.05. SAS version 9.4 was used for statistical analysis. R version 3.5.1 was used to make figures. Data can be accessed at DOI 10.6084/m9.figshare.7746215.

## Results

### Characteristics of the study population

The characteristics of the study population are described in Table 1. A majority of the participants were over 50 years old (66%), and 75% had a BMI less than 25. By design, half of the study participants were active smokers (49%) and 49% were exposed to water arsenic >50 μg/L, the drinking water standard for arsenic in Bangladesh. The levels of arsenic exposure in Bangladesh were quite variable in our study population and ranged from 10–1,116 μg/gCr. There was a good correlation between water arsenic, used for recruiting participants, and total urinary arsenic adjusted for creatinine (UAs/Cr). The Spearman correlation for water and urinary arsenic was r = 0.502 (n = 197, all data) and r = 0.5340 (n = 181, analyzed data), p<0.0001. We used UAs/Cr for association with immune biomarkers because we considered it a more proximal indicator of exposures that occurred at the time of collection of blood for immune assays.

**Table 1:** Demographic, exposure, and biomarker characteristics of Bangladeshi men included in this study population (n = 181).

Variables	Mean (SD)	Median (range)
*Demographics*		
Age	51.7 (6.3)	52 (36, 65)
BMI	22.2 (3.8)	21.5 (13.7, 34.7)
Ever smoked	48.6%	—
*Exposure*		
Water arsenic (ppb)	93.8 (120.9)	44.9 (0.11, 730.0)
PAH-DNA adducts (per/10^8^ nucleotides)	2.21 (1.42)	1.84 (0.47, 8.00)
UAs/Cr (μg/g)	161.5 (180.1)	96.3(10.0, 1116.0)
*Biomarkers*		
Anti-CD3/anti-CD28 (CPM)	101992 (38817)	97980 (15982, 205101)
TNFa (pg/ml)	4639 (2304)	4554 (13.4, 11744)
IFNg (pg/ml)	10018 (8588)	8289 (0.23, 59727)
IL-1b (pg/ml)	152.4 (104.6)	145.2 (2.8, 725.6)
IL-2 (pg/ml)	2810 (1869)	2607 (0.04, 9242)
IL-4 (pg/ml)	25.1 (16.9)	21.4 (0.01, 123.8)
IL-6 (pg/ml)	435.5 (786.6)	296.9 (13.7, 9984)
IL-8 (pg/ml)	27092 (17181)	24204 (2733, 115200)
IL-10 (pg/ml)	274.8 (171.4)	255.6 (0.07, 997.1)
IL-17A (pg/ml)	1912 (1750)	1489 (0.2, 11885)

Note: Counts per minute (CPM) tritiated (^3^H) thymidine incorporation.

### Relationships between the of markers of immune function

Among the ten immune parameters, IL-4 and IL-8 were not correlated; all others were positively correlated with each other (p < .0001). Correlations between the other markers ranged from 0.18 to 0.75, and were statistically significant. Correlation coefficients relating pro-inflammatory cytokines to anti-CD3/anti-CD28 stimulated proliferation were as follows: IL-1b (r = 0.47, p < .0001), IL-6 (0.31, p < .0001) and IL-8 (r = 0.37, p < .0001). Correlations of IL-1b with IL-6 and IL-8 were r = 0.75, p < .0001 and r = 0.56, p < .0001 respectively. The correlation between IL-6 and IL-8 was: r = 0.48, p<0.0001.

### Relationships between urinary arsenic and immune parameters

**Fig 1:**
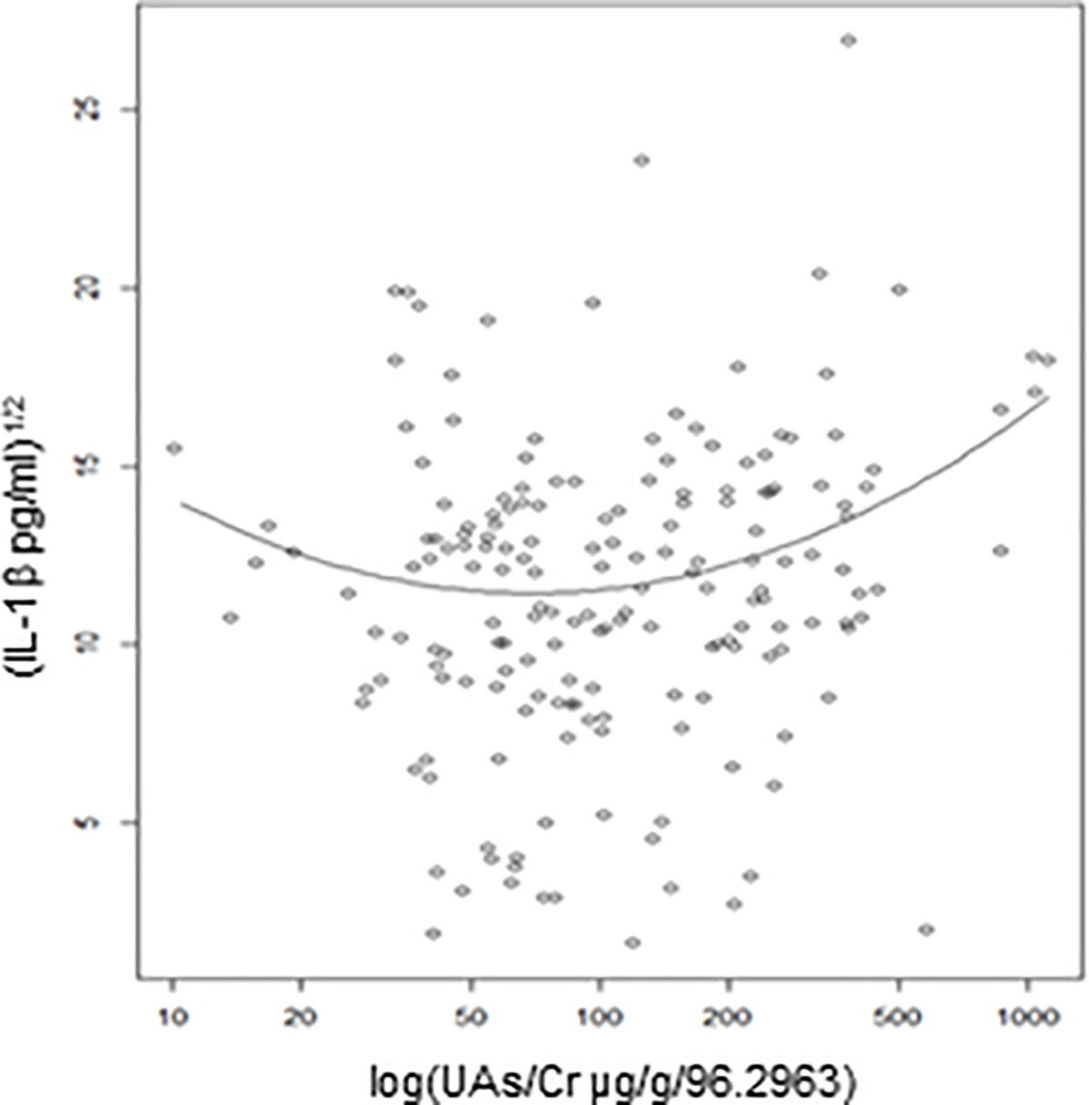
Urinary arsenic (UAs/Cr) is associated with changes in cytokine production. Increase in proinflammatory cytokines; IL-1b (chemokine; growth related oncogene) was associated with increased arsenic exposure (measured by urinary arsenic). Individual data points represent individual observations, solid lines represent estimated regression lines.

Summarized results of multivariable regression analyses for associations between arsenic exposure and the immune parameters are shown in Table 2. We observed strong positive associations between urinary As and IL-1b, IL-2, IL-4 and IL-6 in adjusted models for age, BMI, smoking status and PAH-DNA adducts. The association for IL-8 was marginally significant. However, after controlling for false discovery rate (FDR) in multiple tests, only the association between UAs/Cr and IL-1b (p = 0.01; FDR = 0.05) remained statistically significant IL-1b. As shown in Fig 1, the relationship between IL-1b and UAs/Cr was non-linear.

**Table 2:** Association between urinary arsenic concentration per creatinine (UAs/Cr) and immune parameters^1^.

Immune parameter	DR^2^ (%)	p-value	FDR	B_1_ (95% CI)	B_2_ (95% CI)
Anti-CD3/anti-CD28 (/10,000)	0.06	0.74	0.8	0.1 (-0.54, 0.76)	—
TNFa (/1000)	0.22	0.52	0.66	0.12 (-0.27, 0.52)	—
(IFNg)^1/2^	0.01	0.86	0.86	-0.61(-7.49, 6.27)	—
(IL-1b)^1/2^	5.21	0.01	0.05	0.47 (-0.27, 1.21)	0.71 (0.13, 1.28)
(IL-2)^1/2^	2.24	0.03	0.10	3.25(0.37, 6.14)	—
(IL-4)^1/2^	3.10	0.05	0.12	0.21 (-0.08, 0.49)	-0.26 (-0.48, -0.04)
log(IL-6)	2.50	0.03	0.10	—	0.12 (0.01, 0.24)
(IL-8)^1/3^	1.91	0.06	0.12	—	0.75 (-0.03, 1.53)
(IL-10)^1/2^	0.32	0.42	0.65	0.36 (-0.53, 1.26)	—
(IL-17A)^1/3^	0.27	0.46	0.65	0.23 (-0.37, 0.83)	—

^1^ Adjusted for age, BMI, ever smoked and PAH-DNA adducts (n = 181)

Note: DR^2^: The change in R^2^ for percent of variation in outcome explained by the effect of UAs/Cr adjusting for other variables. B_1_: estimated coefficient of X; B_2_: estimated coefficient of X^2^; for X = log(UAs/Cr /96.2963). CI: Confidence interval;—no relationship found

### Relationships between PAH-DNA adducts and immune parameters

The various patterns in the associations between PAH-DNA adducts and the immune parameters, adjusting for age, BMI, ever smoked and effect of UAs/Cr are shown in Table 3. In contrast to urinary As, we observed strong inhibitory effects of PAH-DNA adduct on immune function 6 immune function parameters namely TCP, IFNg, IL-1b, IL-2, IL-4, IL-10 and IL-17A. Importantly, the relationship between PAH-DNA adducts and all the immune function parameter remained significant (<0.05) following FDR for multiple testing, except for IL-4.

**Table 3:** Association between PAH-DNA adducts and immune parameters^1^.

Immune parameter	DR^2^ (%)	p-value	FDR	B_1_ (95% CI)	B_2_ (95% CI)	B_3_ (95% CI)
Anti-CD3/CD28 (/10,000)	6.55	<0.01	0.01	-0.35 (-1.26, 0.57)	-1.79 (-2.79, -0.79)	—
TNFa (/1000)	0.58	0.31	0.31	-0.27 (-0.81, 0.26)	—	—
(IFNg)^1/2^	13.69	<0.01	<0.01	-50.29 (-70.66, -29.92)	-1.61 (-12.66, 9.44)	26.57 (10.74, 42.40)
(IL-1b)^1/2^	6.51	0.01	<0.01	-3.25 (-5.33, -1.18)	-0.88 (-2.01, 0.25)	1.81 (0.20, 3.426)
(IL-2)^1/2^	17.12	<0.01	<0.01	-21.845 (-30.39, -13.3)	-6.64 (-11.27, -2.00)	12.36 (5.72, 18.99)
(IL-4)^1/2^	3.29	0.04	0.06	-0.23 (-0.61, 0.15)	-0.52 (-0.93, -0.11)	—
log(IL-6)	3.85	0.07	0.08	-0.59 (-1.02, -0.16)	0.0737 (-0.16, 0.31)	0.43 (0.09, 0.77)
(IL-8)^1/3^	2.44	0.10	0.12	-0.37 (-1.81, 1.06)	1.42 (-0.13, 2.97)	—
(IL-10)^1/2^	11.82	<0.01	<0.01	-5.61 (-8.28, -2.95)	-1.513 (-2.96, -0.06)	3.39 (1.32, 5.46)
(IL-17A)^1/3^	13.10	<0.01	<0.01	-3.90 (-5.68, -2.12)	-1.05 (-2.02, -0.09)	1.97 (0.58, 3.35)

^1^ Adjusted for age, BMI, ever smoked and UAs/Cr (n = 181)

Note: DR^2^: The change in R^2^ for percent of variation in outcome explained by the effect of PAH-DNA adducts adjusting for other variables. B_1_: estimated coefficient of X; B_2_: estimated coefficient of X^2^, B_3_: estimated coefficient of X^3^; for X = log(PAH-DNA adducts /1.8357). CI: Confidence interval

**Fig 2:**
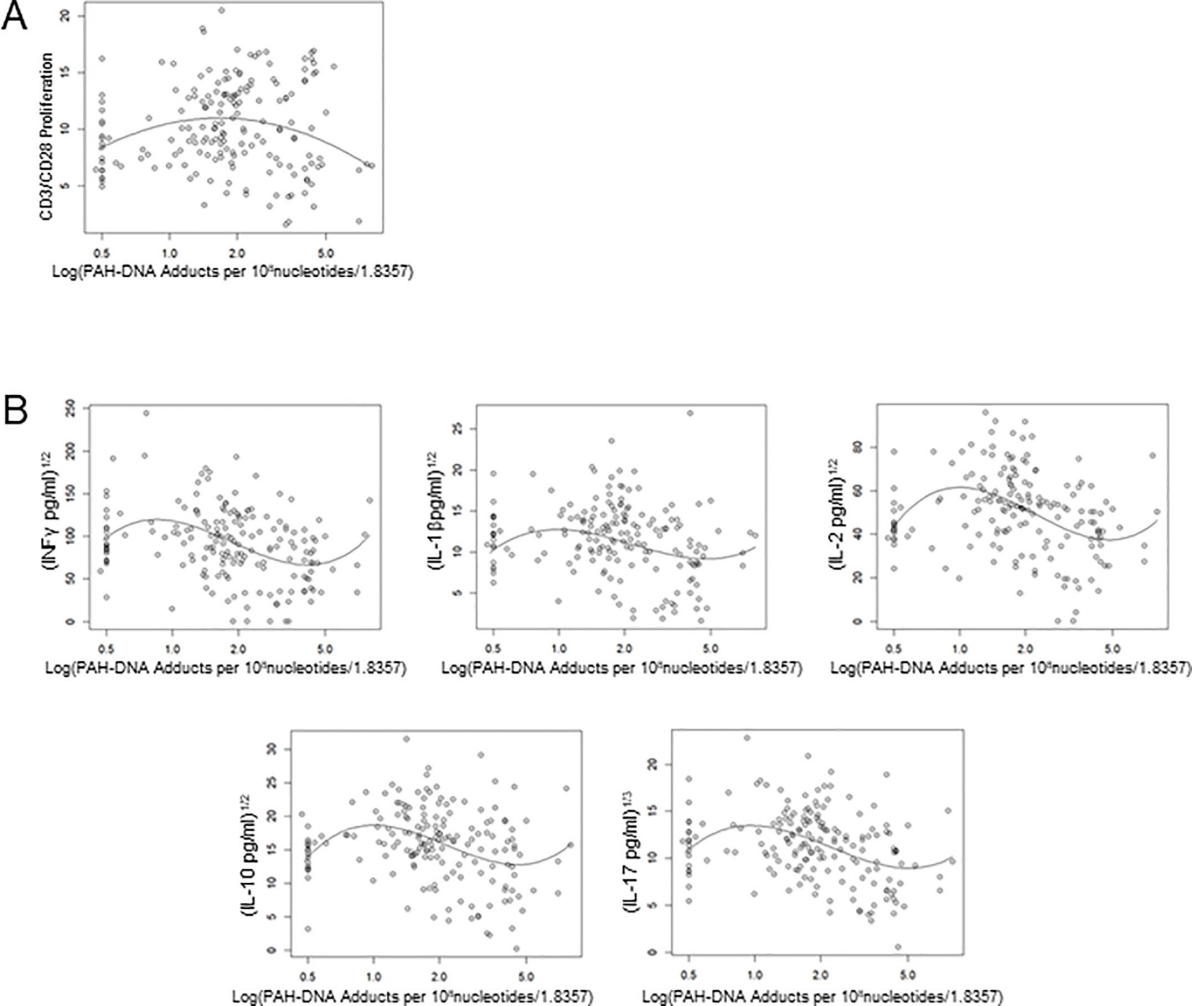
Associations between logPAH-DNA adducts and TCP and cytokine production. Second order, quadratic terms, (A) were used to model the association between logPAH-DNA adducts and anti-CD3/anti-CD28 stimulated TCP. Third order, cubic terms, (B) model the association between PAH exposure and cytokine production (IFNg, IL-1b, IL-2, IL-10 and IL-17A). Individual data points represent individual observations, solid lines represent estimated regression lines.

Fig 2 graphically represents the associations found for these biomarkers. Models with linear and quadratic terms described non-monotonic association between PAH-DNA adducts and anti-CD3/anti-CD28 (Fig 2A), while the models with 3^rd^ -order polynomials described the associations between PAH-DNA adducts and IFNg, IL-1b, IL-2, IL-6, IL-10 and IL-17A. Anti-CD3/anti-CD28 induced TCP had values that increased and then decreased, described as an inverted U shape, with PAH-DNA adducts. The relationship between changes in PAH-DNA adducts and other biomarkers was more complex. For the majority of samples in the 1–4 logPAH-DNA range, IFNg, IL-1b, IL-2, IL-10 and IL-17A showed a trend towards a decrease in these immune parameters.

## Discussion

Despite a significant number of epidemiological studies that have examined the influence of environmental arsenic exposure on cancer [33, 34], cardiovascular disease [6, 35] altered lung function [36], and infection [14, 37–39], there are only a few studies that examined the effects of chronic arsenic exposure on the human immune system. This is somewhat surprising as the immune system in known to play a critical role in protection from infections and immune surveillance of cancer. There have been a few developmental immune system studies in children who received *in utero* exposures to arsenic [26, 27, 37, 40, 41]. These studies focused on T-cell development and responses to childhood immunizations, and generally found decreased responses to childhood MMR and BCG vaccines. In addition, suppression of IL-2 and TNFa was associated with urinary arsenic, and was particularly strong in children with recent infections [27].

In adults and children, previous work by Soto-Pena et al. [24] and Biswas et al. [23] showed that human TCP was suppressed by arsenic in cohorts exposed to arsenic via drinking water. Other human population studies have shown that arsenic modulates both the adaptive [27] and innate [42] immune systems. Numerous studies in HPBMC have shown that human T-cells are particularly sensitive to *in vitro* arsenic exposures [18, 20–22].

In the present study, we obtained fresh blood samples from donors who were seen for routine health checkups as part of the Columbia University and University of Chicago HEALS Cohort health study at a field clinic in Araihazar, Bangladesh [43]. Many donors recruited from this region are known to have high exposures to arsenic in drinking water. Half of the study participants had above and half had below 50 μg/L of arsenic in their well water, the drinking water standard for arsenic in Bangladesh. Overall exposure to arsenic was estimated by measuring urinary arsenic and correcting for hydration status with urinary creatinine. Because we wanted to assess the interaction of PAHs with arsenic, we recruited smokers who might have additional PAH exposure. Accordingly, we recruited approximately equal number of never smokers and smokers for this study. Only males were recruited because the rate of cigarette smoking in Bangladeshi women is extremely low. We used blood PAH-DNA adducts as a marker of PAH exposure. However, we found that PAH-DNA adducts were only marginally higher in smokers than never smokers, possibly because of the relatively ubiquitous exposure to household smoke from the indoor combustion of biomass for cooking.

We performed routine hematology analyses on fresh blood, and found that urinary arsenic was associated with a decline in RBC counts, and many participants had a mild elevation of white blood cell counts [28]. For immune assessments, the present study mostly focused on examining the function of peripheral blood lymphocytes, especially T-cells, as these cells have previously been found to be sensitive to the effects of arsenic exposure. We found no association of urinary arsenic with TCP stimulated by anti-CD3/anti-CD28 antibodies, which differs from previous reports where TCP was inhibited by arsenic [23, 24]. The earlier studies were conducted among children or adults exposed to very high concentrations of arsenic with arsenical skin lesions involving only 38 individuals. The difference in results may be explained by the use of weak mitogens (PHA and Con A) by those investigators. In addition, we carefully controlled for cell viability, which is known to be altered by arsenic. We prefer the use of anti-CD3/anti-CD28 as it is more commonly used today for robust T-cell activation and cytokine studies. Because we used supernatants from the anti-CD3/anti-CD28 stimulated cultures to measure cytokine secretion, we could compare directly with the TCP cultures.

We found several positive associations between UAs/Cr and cytokines (p ≤0.05). However, after adjusting for false discovery rate (FDR) only IL-1b remained statistically significant (Table 2). The relationship with arsenic exposure is quite complex. The non-monotonic association was modeled by including quadratic terms. The model indicates decreased production or no significant change at low concentrations of arsenic and increased IL-1b production at high arsenic concentrations.

An analysis of the association of PAH-DNA adducts with immune parameters showed that PAH exposure was strongly linked to inhibition of TCP, a result that we have previously reported following *in vitro* exposures of HPBMC [44].The relationship between PAH-DNA adducts and cytokine production was again found to be complex, with low levels of adducts being associated with increases in IFNg, IL-1b, IL-2, IL-6, IL-10, and IL-17A (Fig 2). However, high PAH-DNA adduct levels were associated with a decrease in all of these cytokines. A formal statistical test of PAH-DNA adducts and UAs/Cr levels showed no interactions between these two exposures. This was somewhat surprising because we have previously shown in mice a PAH and arsenic interaction *in vitro* [17, 45] and in *in vivo*. However, these studies were performed using mouse peripheral lymphoid tissues (spleen and thymus). We cannot assume that the findings would be comparable in human blood cells. In addition, the clearest indication of synergistic interactions between PAHs and arsenic was in mouse thymus *in vivo* and *in vitro* studies, whereas we examined DNA damage and PARP inhibition [45]. Genotoxicity has been associated with cancer induced by arsenic and PAHs [46]. In epidemiological studies, cigarette smoking is associated with an increase in arsenic induced urothelial bladder tumors [47]. Therefore, our conclusion is that immune function, as measured by TCP and cytokine production in HPBMC, is independently associated with PAHs and arsenic (Table 4). Our immune endpoints are likely not related to genotoxicity.

**Table 4:** Summary of findings.

Immune Parameter	Associated with UAs/Cr	Associated with PAH-DNA Adducts	Arsenic–PAH Interaction
T-cell Proliferation (TCP) (anti-CD3/anti-CD28)	No	Yes	No
TNFa	No	No	No
IFNg	No	Yes	No
IL-1b	Yes	Yes	No
IL-2	No	Yes	No
IL-4	No	No	No
IL-6	No	No	No
IL-8	No	No	No
IL-10	No	Yes	No
IL-17A	No	Yes	No

In summary, in a Bangladeshi healthy male cohort we found complex relationships between immune effects measured in HPBMC *ex vivo* following exposures to environmental arsenic and PAHs. Increased arsenic exposure was associated with increased proinflammatory cytokine production of IL-1b. In contrast, the associations between PAH-DNA adducts and cytokines appeared to be biphasic, with low exposures associated with an increase in proinflammatory cytokines and higher exposures with a decrease in these cytokines. There was no interaction between arsenic and PAH exposure in terms of immune modulation. It is notable that the immune parameters measured in these studies are associated with non-genotoxic pathways, such as cell signaling, and therefore genotoxic pathways and endpoints may need to be measured to further assess the interactions of PAHs and arsenic in humans. Ongoing studies are assessing the effects of arsenic on lung function and their association with immune endpoints in males and females.
